# Giant Magnetic Band Gap in the Rashba-Split Surface State of Vanadium-Doped BiTeI: A Combined Photoemission and *Ab Initio* Study

**DOI:** 10.1038/s41598-017-03507-0

**Published:** 2017-06-13

**Authors:** I. I. Klimovskikh, A. M. Shikin, M. M. Otrokov, A. Ernst, I. P. Rusinov, O. E. Tereshchenko, V. A. Golyashov, J. Sánchez-Barriga, A. Yu. Varykhalov, O. Rader, K. A. Kokh, E. V. Chulkov

**Affiliations:** 1 Saint Petersburg State University, 198504 Saint Petersburg, Russia; 2 Tomsk State University, 634050 Tomsk, Russia; 3 Donostia International Physics Center (DIPC), 20018 San Sebastián/Donostia, Basque Country, Spain; 4 Departamento de Física de Materiales UPV/EHU, Centro de Física de Materiales CFM - MPC and Centro Mixto CSIC-UPV/EHU, 20080 San Sebastián/Donostia, Basque Country, Spain; 5 A.V. Rzhanov Institute of Semiconductor Physics, 630090 Novosibirsk, Russia; 6 Novosibirsk State University, 630090 Novosibirsk, Russia; 7 V.S. Sobolev Institute of Geology and Mineralogy, 630090 Novosibirsk, Russia; 8 Max Planck Institute of Microstructure Physics, Weinberg 2, 06120 Halle, Germany; 9 Helmholtz-Zentrum Berlin für Materialien und Energie, Elektronenspeicherring BESSY II, Albert-Einstein-Strasse 15, 12489 Berlin, Germany; 10 Institut für Theoretische Physik, Johannes Kepler Universität, A 4040 Linz, Austria

## Abstract

One of the most promising platforms for spintronics and topological quantum computation is the two-dimensional electron gas (2DEG) with strong spin-orbit interaction and out-of-plane ferromagnetism. In proximity to an *s*-wave superconductor, such 2DEG may be driven into a topologically non-trivial superconducting phase, predicted to support zero-energy Majorana fermion modes. Using angle-resolved photoemission spectroscopy and *ab initio* calculations, we study the 2DEG at the surface of the vanadium-doped polar semiconductor with a giant Rashba-type splitting, BiTeI. We show that the vanadium-induced magnetization in the 2DEG breaks time-reversal symmetry, lifting Kramers degeneracy of the Rashba-split surface state at the Brillouin zone center *via* formation of a huge gap of about 90 meV. As a result, the constant energy contour inside the gap consists of only one circle with spin-momentum locking. These findings reveal a great potential of the magnetically-doped semiconductors with a giant Rashba-type splitting for realization of novel states of matter.

## Introduction

Two-dimensional electron systems have been attracting the researchers interest for many years due to a number of unique electronic effects. A classical example is the quantum Hall effect^1^ – a phenomenon of simultaneous transverse resistance quantization and longitudinal resistance drop, arising in a high-mobility two-dimensional electron gas (2DEG) under a strong magnetic field. After this discovery it was realized that a similar behavior can be achieved without an external field in materials with a strong spin-orbit interaction (SOI), and the quantum *spin* ^2^ and *anomalous* ^3^ Hall effects were predicted to occur in the 2D paramagnetic and ferromagnetic (FM) insulating systems, respectively. These and other intriguing findings^4–7^ have established a field where the spin-orbit and magnetic effects interplay is aimed at promising applications in spintronics and quantum computing^4, 8–12^.

Of particular interest is the SOI effect on the 2DEG systems with broken inversion symmetry, manifesting itself in the so-called Rashba splitting of the electronic states^13^. It has been predicted that, being proximitized to a magnetic insulator on the one hand and to an *s*-wave superconductor on the other, a semiconductor thin film with a Rashba splitting may undergo a peculiar phase transition and turn into a topological superconductor^6^. According to the proposal, a proximity of magnetic insulator to the semiconductor breaks the time-reversal symmetry (TRS) whereby an exchange gap is opened at the crossing point of the two Rashba-split bands, creating a single-circle Fermi contour. The states with opposite spins at opposite momenta of the contour can be paired *via* the proximity to an *s*-wave superconductor, which would gap the spectrum globally. This situation can lead to a topological superconductor phase, expected to host exotic quasiparticles, the Majorana fermions^14^. Their incarnation in a solid state device would significantly advance quantum computation and, having the suitable experimental setups theoretically proposed^6, 15–17^, several studies have been performed in an effort to probe them^18–21^.

As is seen from the foregoing, the design of a playground for the Majorana fermions realization involves choices of the Rashba semiconductor and the way of its magnetic functionalization. In the experiments performed recently^18–20^, the latter is done using an external magnetic field rather than the magnetic proximity effect. However, for application purposes, an external field needs to be substituted by an intrinsic one^22^. This can also be achieved by a magnetic doping of a Rashba semiconductor. In fact, this approach has proven successful in the realization of the quantum anomalous Hall state. It has been observed in magnetically-doped topological insulators^23^ – systems where an out-of-plane magnetization breaks the TRS and thus opens a gap in the topological surface state^24, 25, 26^. On the other side of the system’s design problem is the choice of the Rashba semiconductor. To date, the highest known Rashba-type splitting has been reported for the polar semiconductor BiTeI^27–31^. The figure-of-merit of the Rashba splitting, *E*_R_, being the energy separation between the crossing point and parabolic minimum, reaches 100 meV in this system. Combination of this extraordinary Rashba splitting with a sizable exchange gap would ideally satisfy the requirements suggested for the appearance of the Majorana modes^6^. Here we show that such a state can indeed be achieved in magnetically-doped BiTeI. Using angle-resolved photoemission spectroscopy (ARPES) we show that the vanadium doping of BiTeI lifts the Kramers degeneracy of its Rashba-split Te-terminated surface state at the Brillouin zone (BZ) center resulting in formation of the gigantic exchange gap of approximately 90 meV. The evanescence of the gap with temperature indicates its largely magnetic origin, which, as is suggested by our density functional theory calculations, may stem from the interplay of the V magnetic moments with those of point defects.

**Figure 1:**
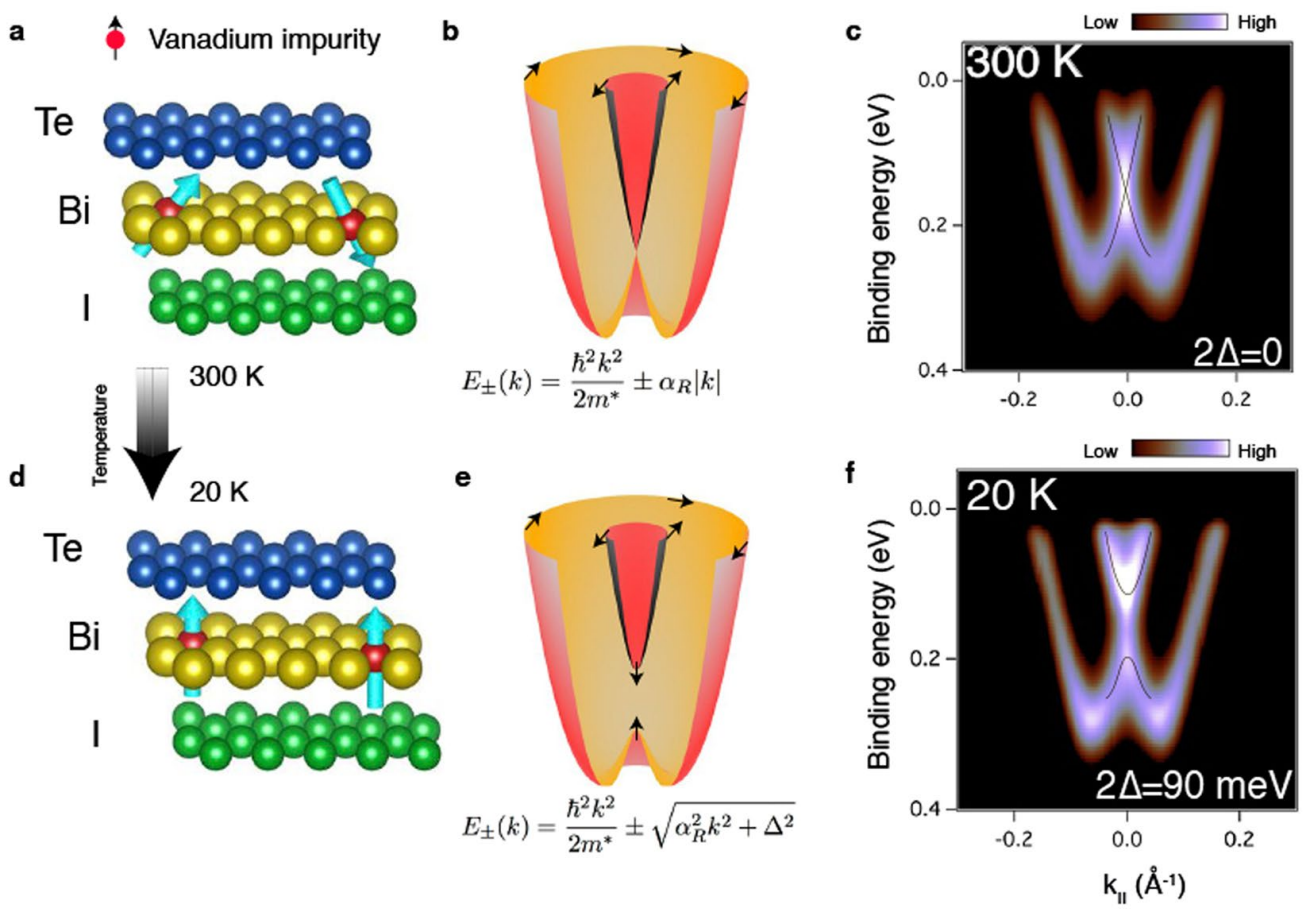
Magnetism and electronic structure of V-doped BiTeI at high and low temperature: schematics and measurements. (**a**) Sketch of the BiTeI TL structure with built-in V atoms and their magnetic moments disordered at high temperature. (**b**) Model dispersion relations of the spin-orbit split 2DEG state in a high temperature paramagnetic state, with *α*_*R*_ being the Bychkov–Rashba coefficient. (**c**) Measured dispersion of the Rashba-split Te-terminated surface state of Bi_0.985_V_0.015_TeI at room temperature. (**d**–**f**) The same set as in (**a**–**c**), but for the ordered magnetic moments and a magnetic gap of 2Δ.

The BiTeI system consists of triple layer (TL) blocks, –Te–Bi–I–, stacked along the hexagonal axis with a van der Waals bonding acting between them. The crystal structure of a single, V-doped BiTeI TL block is schematically shown in Fig. 1a, V atoms being incorporated in the Bi layer. As is depicted in the figure, at high temperature their magnetic moments are expected to be disordered and the TRS preserved. The dispersion relation for the V-doped BiTeI 2DEG state at the Te-terminated surface is in this case described by the two electron-like parabolas, split in a Rashba manner (Fig. 1b), pretty much as in the undoped BiTeI case. Consistently with this picture, the ARPES map for Bi_0.985_V_0.015_TeI measured at 300 K reveals an intensity maximum at the $\bar{{\rm{\Gamma }}}$-point (i.e. *k*_||_ = 0 Å^−1^) and a binding energy ${E}_{b}\simeq 0.15$ eV, indicative of the intersection of the parabolas, i.e. the Kramers degeneracy (Fig. 1c).

Assuming an onset of a magnetically ordered state at low temperatures, one may expect the behavior of the Bi_0.985_V_0.015_ TeI 2DEG state to change significantly upon cooling down the sample. For instance, if the V magnetic moments were ordered ferromagnetically within the topmost TL and the direction of the moments was perpendicular to the surface (see Fig. 1d), the TRS would be broken and the Kramers degeneracy at the $\bar{{\rm{\Gamma }}}$-point would be lifted. This behavior can be described by a Hamiltonian that includes the Bychkov–Rashba and exchange terms,

1 $$H=\frac{{\hslash }^{2}{k}^{2}}{2{m}^{\ast }}+{\alpha }_{R}({{\rm{e}}}_{{\rm{z}}}\times {\rm{k}})\sigma +\lambda {m}^{z}{\sigma }_{z},$$

where *m* ^*^, *α*_*R*_ = 3.8 eVÅ, and *λ* are the effective electron mass, Rashba coefficient for the undoped BiTeI^27^, and the strength of the exchange interaction, respectively, while *m* ^*z*^ is the magnetization, provided by the V atoms. The resulting dispersion relation is then given by

2 $${E}_{\pm }(k)=\frac{{\hslash }^{2}{k}^{2}}{2{m}^{\ast }}\pm \sqrt{{\alpha }_{R}^{2}{k}^{2}+{{\rm{\Delta }}}^{2}},$$

with Δ = *λm* ^*z*^ defining the halfwidth of the local gap, which appears between the inner and outer parts of the Rashba-split state, as shown in Fig. 1e. It is precisely this scenario that develops at the Bi_0.985_V_0.015_ TeI surface after a cooling down to 20 K, as is evidenced by ARPES measurements reported in Fig. 1f, where the appearance of a giant gap of about 90 meV is clearly seen in the $\bar{{\rm{\Gamma }}}$-point.

**Figure 2:**
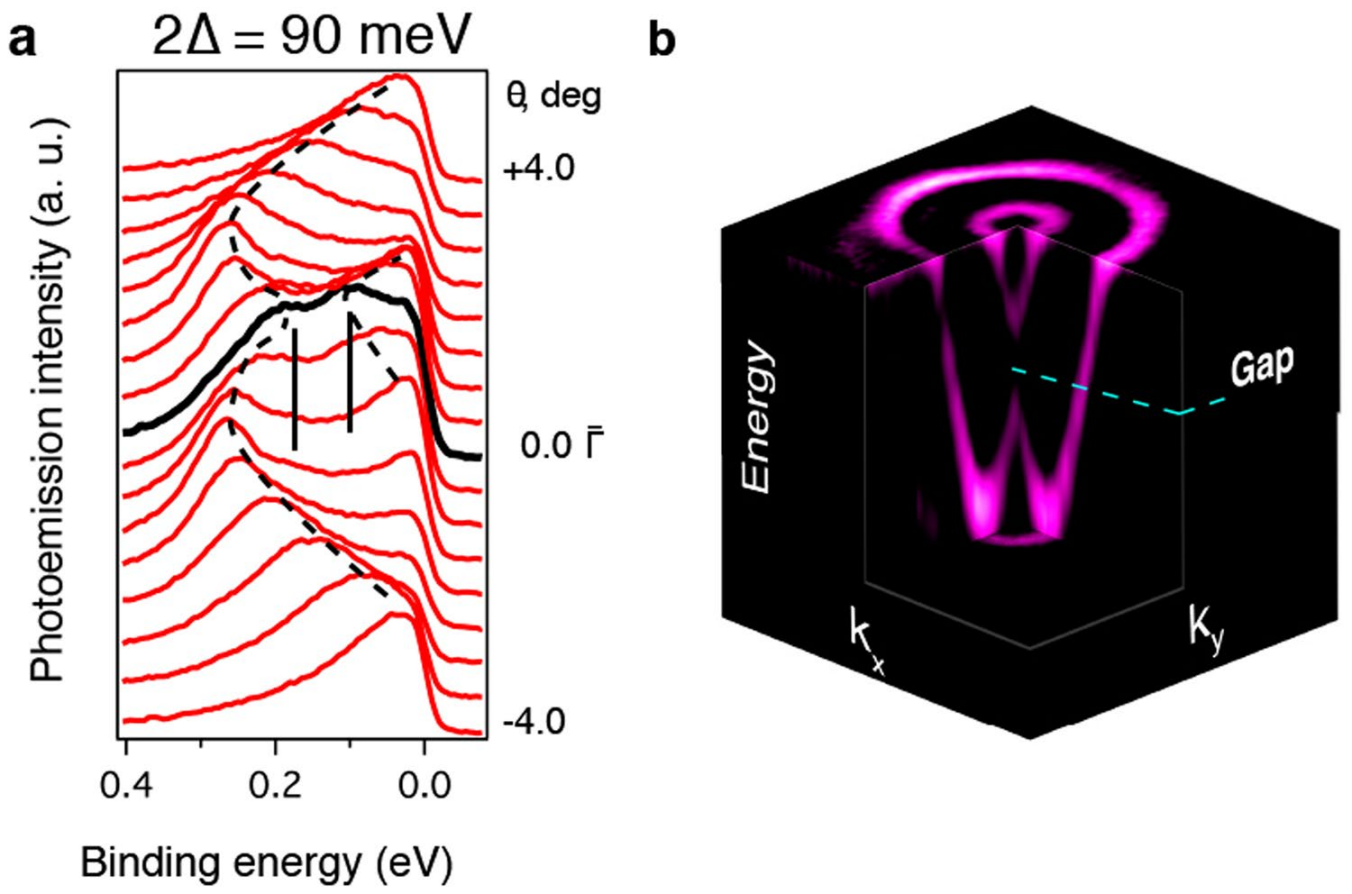
Detailed insight into the exchange gap spectral structure. (**a**) Raw ARPES data for Bi_0.985_V_0.015_ TeI taken at 20 K, extracted from the data presented in Fig. 1f. (**b**) Three-dimensional dispersion relation of the Bi_0.985_V_0.015_ TeI Rashba-split exchange-gapped surface state at 20 K (the second derivative is shown).

Raw ARPES spectra taken for different emission angles at 20 K are shown in Fig. 2a. Far from the $\bar{{\rm{\Gamma }}}$-point, an undisturbed Rashba-like behavior is observed. However at the $\bar{{\rm{\Gamma }}}$-point the spectrum shape is seen to contain the two peaks, corresponding to the exchange gap edges. Based on the comparative analysis of the fitted energy and momentum distribution curves we estimate the gap size as 90 ± 10 meV. It should be noted that, as we show in the Supplementary Information, the intensity within the gap is non-zero owing to the line width tails of the broadened intensity peaks.

The full three-dimensional dispersion relation *E*(*k*_*x*_, *k*_*y*_) is shown in Fig. 2b, where the second derivative of *N*(*E*) is presented. Although the second derivative data cannot be used as an evidence of the gap formation, the overall behavior of the bands is visualized better in this case. Indeed, one can clearly see that within the gap the constant energy contour consists of only one circle, without any feature at/around the $\bar{{\rm{\Gamma }}}$-point.

Thus, the results of our ARPES measurements unambiguously point at largely magnetic origin of the Kramers degeneracy lifting, which takes place only at low temperatures. This implies that, with a decrease of temperature, Bi_0.985_V_0.015_ TeI experiences a transition from the TRS-preserving paramagnetic state to the magnetically-ordered state breaking it. To determine magnetic ordering of the V-doped BiTeI we have computed the exchange coupling parameters and magnon spectra using density functional theory (see Supplementary Information). Our calculations show that although each TL of the V-doped BiTeI is ferromagnetic on its own, the ordering between the adjacent TLs rapidly disappears with the increase of temperature. This, however, does not impede formation of the magnetically ordered state at the surface since the topmost TL is ferromagnetic. These results indicate that it is the topmost FM triple layer that is responsible for the TRS breaking and giant exchange gap opening at the V-doped BiTeI surface.

**Figure 3:**
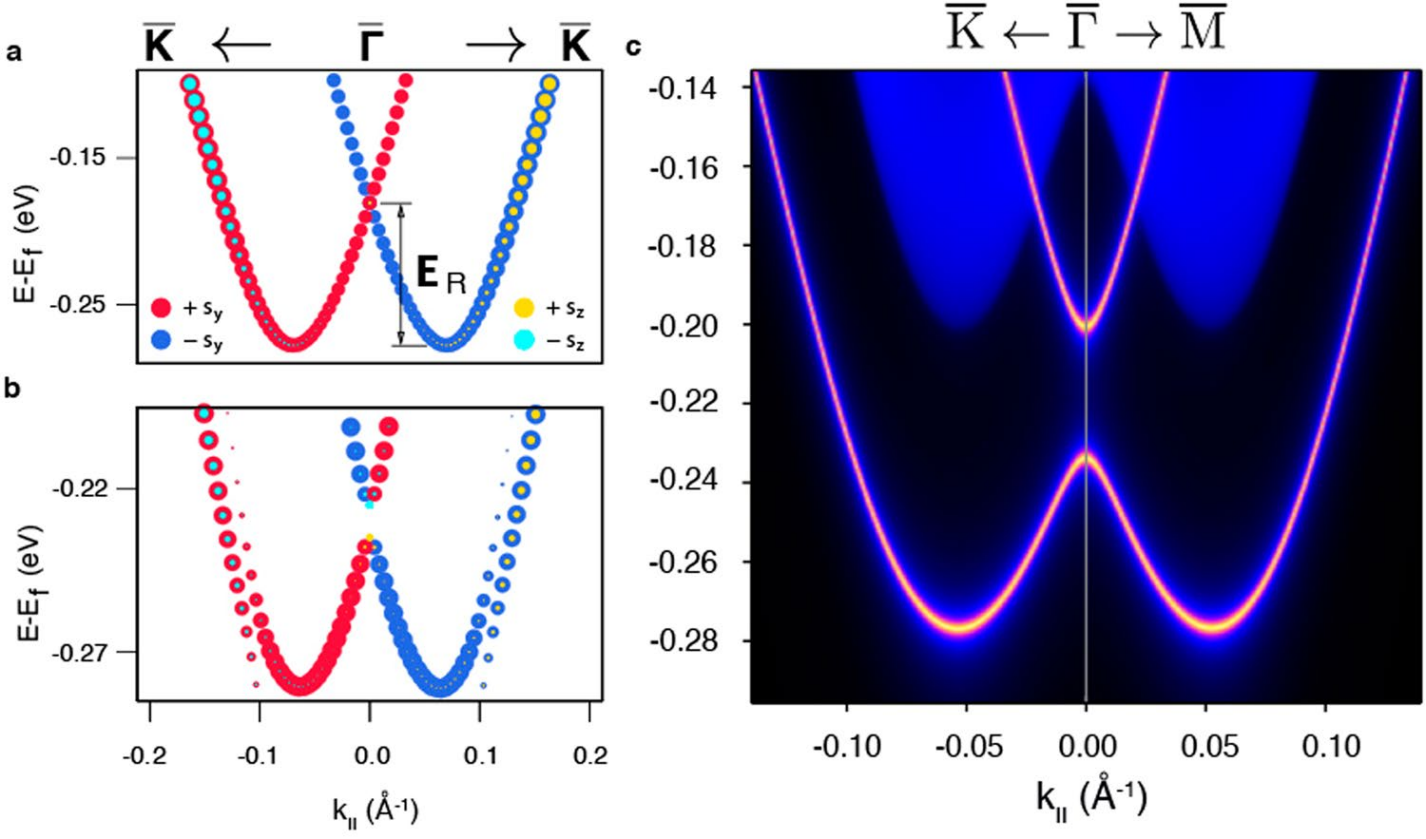
*Ab initio* and tight-binding calculations of pure and V-doped BiTeI. (**a**) Spin-resolved DFT-calculated (0001) surface band structure of pure BiTeI, projected onto the Te-terminated surface TL block. (**b**) The same as (**a**), but for the V-doped BiTeI case. The size of the circles reflects the module of the respective spin projection in each *k*-point. Note, that in (**a**) *E*_*F*_ is as calculated for the H-passivated slab (see Methods Section). For the clear comparison, in (**b**) we shift *E*_*F*_ to the position corresponding to the undoped case. The artificial discontinuity of the outer branches of the Rashba-split state in **b** (~|0.1| Å) stems from the crossing with the quantum well state localized in the subsurface TL (see Supplementary Information). (**c**) Tight-binding calculated surface bandstructure of V-doped BiTeI in the presence of point defect (see Text).

In order to get deeper insight into the giant exchange gap formation we have performed first-principles surface electronic structure calculations. Since only the magnetism of the topmost TL is essential, we only introduce the dopant into the subsurface Bi layer (see Methods section), which allows us to perform calculations in the low concentration limit. Figure 3a shows the Rashba-split surface state of the Te-terminated undoped BiTeI(0001). It is characterized by a slightly smaller *α*_R_ than is typically reported in DFT studies^30, 32^, which is a consequence of a big in-plane supercell choice. However, this is not expected to affect the results and conclusions of the present work (see Supplementary Information). Then, we study the effect of vanadium doping on the Te-terminated BiTeI(0001) surface band structure. One might indeed expect the breakdown of the TRS and, consequently, lifting of the surface state degeneracy at the $\bar{{\rm{\Gamma }}}$-point since, being embedded in the Bi layer, a V atom features a magnetic moment of 2.88 *μ*_*B*_. Note, that the gapping of the surface states as a consequence of the TRS breaking often assumes induction of the spin polarization on the atoms of a nonmagnetic host by different magnetic agents^33, 34^. In the case of V-doped BiTeI, the magnetic moments are mainly induced on Te and I atoms, which are located in the immediate vicinity of the V one and have their *p*-states hybridized with the *d*-states of the latter. This leads to a lifting of the Rashba-split surface state degeneracy at the 2D BZ center, i.e. to an opening of the exchange gap (Fig. 3b). Surprisingly, the magnitude of the calculated gap of 10 meV turns out to be significantly smaller than that of the measured one.

This means that other factors must be contributing to the formation of the giant exchange gap in the Rashba-split surface state. One of these might be a presence of point defects in the V-doped BiTeI. As a recent scanning tunneling microscopy study shows, there is a large number of point defects seen at both the Te- and I-terminated (0001) surface of the pure BiTeI^35^. These defects were suggested to be either vacancies or antisites. We suppose that V-doped BiTeI demonstrates a similar situation as long as the dopant concentration is small, which is the case for our samples having rather low V content (0.5 at.%). Therefore, assuming the coexistence of V atoms with point defects, we have separately considered the magnetism of all possible antisites as well as the vacancies in each atomic layer, that have been placed in the topmost Te-terminated TL of the surface of V-doped BiTeI. It turned out that among all the point defects considered, only a Bi vacancy directly induces the host magnetization (mainly on Te atoms) beyond that caused by the V moments. Moreover, even in the V absence it can supply up to 1.55 *μ*_*B*_ per a vacancy – a case which is by 7 meV more favorable than the one with a suppressed magnetization around a vacant site. Our exchange coupling constants calculations show that the magnetic moments appearing on the Te atoms that surround Bi vacancies tend to align antiparallel to those of V atoms, rather insensitively to the dopant-vacancy distance (the coupling strength is distance-dependent, though). The Te atoms directly bound to the V one are magnetized antiparallel to its local moment as well. Thus, both V atoms and Bi vacancies lead to the enhancement of the overall magnetization of the Te layer. It should be noted, however, that the Bi vacancies do not create any magnetic order in the absence of vanadium. On the other hand, their presence increases somewhat the vanadium local moments and enhances the strength of the exchange interaction between them slightly elevating the Curie temperature. Importantly, V doping can facilitate appearance of the Bi vacancies, since their formation energy gets reduced by approximately 0.23 eV in the dopant vicinity.

To see how the additional magnetization induced by the presence of point defects influences the magnitude of the exchange gap, we have performed band structure calculations with both V and Bi_*vac*_ located in the subsurface atomic layer of BiTeI(0001). However, the results obtained do not allow to unambiguously determine the size of the exchange gap since its edges are broadened due to the Bi_*vac*_ presence (see Supplementary Information). Therefore, to scrutinize the effect of the coexistence of the V dopants and point defects, we resort to a tight-binding approach (see Methods section), where instead of including defects directly, we rather take into account the magnetization they supply. This has been done by introducing an additional Zeeman-like contributions to the Hamiltonian, whose values have been set to the exchange splittings of the Bi, Te and I *p*-states calculated *ab initio* in the presence of both V dopant and Bi vacancy. The validity of the approach employed has been verified against the DFT result shown in Fig. 3b and the tight-binding description has yielded a gap of similar magnitude (11 meV).

Figure 3c shows the result of a tight-binding calculation, performed for the case of the V-doped BiTeI in the presence of Bi_*vac*_. It can be seen that the exchange gap size gets increased appreciably upon accounting for an additional magnetization due to the vacant sites and amounts to 34 meV. This result shows the crucial role, which can play the point defects in the V-doped BiTeI. Note that in real samples the magnetic interplay of point defects and V dopants is probably more intricate than it is theoretically exemplified here. Indeed, in *ab initio* calculations we have only considered simple point defects. For example, a Bi vacancy has been created by a complete removal of one Bi atom from the cell, and not by its relocation to an interstitial or a surface. Similarly, an antisite atom *A* has been introduced in a layer *B* (*A*_*B*_) without leaving behind a vacancy in the layer *A* or creating the *B*_*A*_ antisite which would correspond to the site exchange between atoms *A* and *B*. However, in the experimental situation the defects may be complex, as, e.g., a vacancy-antisite pair, proposed for the undoped BiTeI(0001) surface^35^. Moreover it is not excluded that other complex defects, which have not been observed for the undoped BiTeI case are enabled to form due to V doping. Therefore, further specific and detailed studies, such as scanning tunneling microscopy measurements and simulations, combined with careful theoretical characterization of the observed dopant-defect complexes, are required to uncover missing factors contributing to the formation of the giant exchange gap in the Rashba-split surface state. There is, however, a fundamental reason why the calculated exchange gap size is smaller than the experimentally measured one. The stationary density functional theory is not designed to describe excited state properties and typically underestimates the band gap sizes by 30–70% of the experimental ones^36^.

Lately, the issue of the Kramers degeneracy lifting observed for the surface states of different bulk-doped systems is under intense discussion^9, 24, 37–39^. Noteworthy, a 2DEG Rashba-split state with an exchange gap of 90 meV has recently been observed at the SrTiO_3_ surface^38^, however the origin of the strong Rashba-like splitting in this system is not clear yet^40^. In the case of impurity-doped topological insulators, the Kramers point splitting was experimentally observed^9, 24^, even at room temperature^37, 39^. The explanations involving appearance of the superparamagnetic clusters^37^ or a strong resonant scattering due to the dopant in-gap states^39^ have been put forward. The latter case is particularly interesting since it may lead to the surface state gapping without magnetism if a sufficiently strong potential is created by the impurities^41^. However, this effect would not depend on temperature and, besides, such a perturbation would gap the surface state not only in the $\bar{{\rm{\Gamma }}}$-point, but at finite *k* as well. The results of our ARPES measurements exclude such a scenario, since the gap is seen only in the $\bar{{\rm{\Gamma }}}$-point and disappears with the increase of temperature. Another contribution may possibly come from the photoemission process during ARPES measurements of Bi_0.985_V_0.015_TeI. Photoexcitation of electrons in V-doped BiTeI by ultraviolet light can induce the spin accumulation at the surface owing to giant spin-orbit interaction. Similar effects had been observed in BiTeI in Refs 31, 42 by laser excitation. Spin accumulation acts like effective magnetic field, and thus, can result in enhancement of the value of the exchange gap at the $\bar{{\rm{\Gamma }}}$ point.

## Conclusions

In summary, by means of ARPES we have shown that the giant-Rashba-split electronic state residing at the Te-terminated surface of the vanadium-doped polar semiconductor BiTeI exhibits a huge band gap at the Brillouin zone center. Our photoemission measurements, complemented by detailed theoretical calculations, allow to conclude that the origin of this gap largely lies in the time-reversal symmetry breaking, that stems from the magnetically ordered state, onsetting at the vanadium-doped BiTeI surface at low temperature. With this finding, magnetically doped BiTeI poses itself as a promising material to be combined with *s*-wave superconductors with the objective of Majorana fermion formation.

## Methods

Single crystals of BiTeI + 0.5% V were grown using 99.999% pure powders of Bi, Te, I and V by the Bridgman method. ARPES experiments were carried out at Helmholtz-Zentrum Berlin (BESSYII) at beamlines UE112-SGM with the assistance of a Scienta R4000 energy analyzer using synchrotron radiation with the energy of 23 eV. This photon energy allows to obtain the photoelectrons mainly from the surface layers and to avoid the resonant effects appearing due to the Bi 5*d* levels excitation. The overall energy resolution for the ARPES measurements was 5 meV. Samples were cleaved *insitu* at the base pressure of 6 × 10^−11^ mbar. In order to check the contamination of the surface, the cooling down to 20 K had been done both before and after the cleavages. Part of the experiments were performed out in the Research Resource Center “Physical methods of surface investigation” of Saint Petersburg State University. The crystalline order and cleanliness of the surface were verified by low energy electron diffraction (LEED) and X-ray photoelectron spectroscopy (XPS).

*Ab initio* calculations were performed using the density functional theory in the generalized gradient approximation to the exchange-correlation potential^43^. The localized V 3*d*-states were described in the framework of the GGA + *U* approach^44^, with an effective *U* − *J* value of 3 eV (Dudarev’s scheme^45^). Surface electronic structure calculations were performed within the projector augmented-wave method^46^ in the VASP implementation^47, 48^. The Hamiltonian contained the scalar relativistic corrections and the spin-orbit coupling was taken into account by the second variation method^49^. In order describe the van der Waals interactions we made use of DFT-D2 approach proposed by Grimme^50^. We set the energy cutoff for the plane-wave expansion of wave functions to be equal to 250 eV and chose a $\bar{{\rm{\Gamma }}}$-centered *k*-point grid of 3 × 3 × 1 to sample the two-dimensional Brillouin zone. The (0001) surface of V-doped BiTeI was simulated by a 3TL-thick slab and (3 × 3) hexagonal in-plane supercell with 9 atoms per single layer. Vanadium atom and/or Bi vacancy were placed in the surface TL, which is terminated by Te atoms. The doping of the subsurface TL wasn’t considered since the Rashba-split state at Te-terminated BiTeI(0001) resides predominantly in the topmost TL block. Thus the V concentration in our calculations was of about 1.23%. Upon introduction of V atom and Bi vacancy, a structural optimization was performed using a conjugate-gradient algorithm and a force tolerance criterion for convergence of 0.05 eV/Å. Vanadium’s magnetic moment was directed out-of-plane, i.e. perpendicular to the surface. As far as the iodine surface is concerned, it was passivated by placing a hydrogen atom on top of each iodine one. All calculations were performed using a model of repeating slabs separated by a vacuum gap of a minimum of 10 Å.

Magnetic ordering of the V-doped BiTeI was studied using the Korringa-Kohn-Rostoker method^51, 52^ within a full potential approximation to the crystal potential^53^. Final concentration of the V atoms and Bi vacancies was accounted for using the coherent potential approach as it is implemented within the multiple scattering theory^54^. We took an angular momentum cutoff of *l*_*max*_ = 3 for the Green’s function and a k-point mesh of 50 × 50 × 50 (50 × 50 × 1) for the 3D (2D) Brillouin zone integration.

*Ab-initio*-based tight-binding calculations were performed using the VASP package with the WANNIER90 interface^55, 56^. The wannier basis chosen consists of six spinor *p*-type orbitals $|{p}_{x}^{\uparrow } >$, $|{p}_{y}^{\uparrow } >$, $|{p}_{z}^{\uparrow } >$, $|{p}_{x}^{\downarrow } >$, $|{p}_{y}^{\downarrow } >$, $|{p}_{z}^{\downarrow } >$ for each atom, while the low-lying *s* orbitals were not taken into consideration. The band-bending potential was obtained from previous DFT calculations^57^. A Zeeman term in Eq. (2) was set to the exchange splitting of the Bi, Te and I *p*-states, as calculated within a scalar-relativistic approach. The surface band structure was calculated for the semi-infinite system within the Green function approach^58, 59^.

## Electronic supplementary material

Supplementary Information
