# Supplementary material for: Giant Magnetic Band Gap in the Rashba-Split Surface State of Vanadium-Doped BiTeI: A Combined Photoemission and Ab Initio Study
Source: Sci Rep. 2017 Jun 13;7:3353. doi: 10.1038/s41598-017-03507-0 (PMC5469768; doi:10.1038/s41598-017-03507-0)
Supplement: Supplementary file 1 — Supplementary Information [file 41598_2017_3507_MOESM1_ESM.pdf]

Supplementary Information

Giant Magnetic Band Gap in the Rashba-Split Surface State of Vanadium-Doped BiTeI: a Combined Photoemission and Ab Initio Study

I. I. Klimovskikh, A.M. Shikin, M. M. Otrokov, A. Ernst, I. Rusinov, O. E. Tereshchenko, V. Golyashov, J. Sánchez-Barriga, A. Yu. Varykhalov, O. Rader, K. A. Kokh, and E. V. Chulkov

Supplementary Note 1: Characterization of the V-doped BiTeI surface

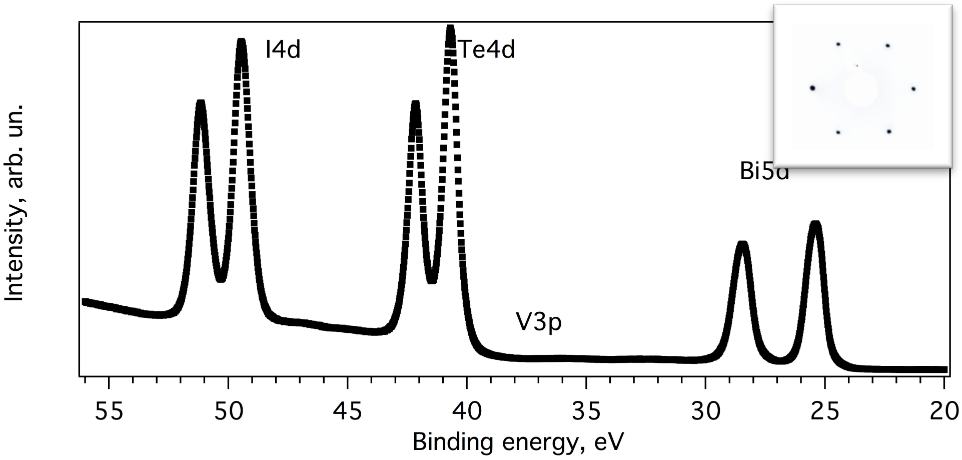

Fig. 1S XPS spectrum of $Bi_{0.985}V_{0.015}TeI$ taken at a photon energy of 120 eV. According to Ref. [SR1], the binding energies of the Bi, Te and I peaks correspond to the Te-terminated surface without mixing with I-terminated terraces. LEED pattern, taken at the energy of primary electrons of 62 eV, is shown in the inset.

According to a recent study performed on the pristine BiTeI(0001) [SR1], after a cleavage the BiTeI surface features two distinct terminations – the Te and I ones, showing different electronic structure. We have studied the Te-terminated surface which is known to show the electron-like Rashba-split surface state (see Fig. 1c of the manuscript). Besides, the core levels binding energies are different for the terraces with different atomic termination. Fig. 1S presents a core level photoemission spectrum, with the binding energies of the I and Te 4*d* states, as well as the Bi 5*d* states well corresponding to those reported for the BiTeI Te-terminated surface [SR1]. Note, that intensity of the V 3*p* peak is at the noise level, since the V concentration is small (0.5 at. %).

A LEED pattern, presented in the inset to Fig. 1S, shows the usual hexagonal structure without any extra reflexes or superstructure.

Supplementary Note 2: ARPES intensity analysis of the gap

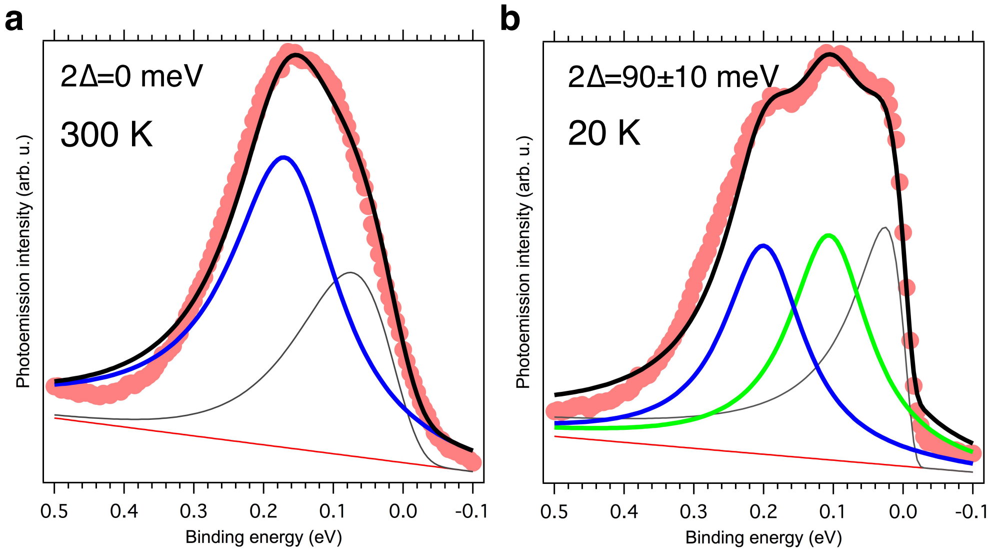

Fig. 2S Photoemission spectra of $Bi_{0.985}V_{0.015}TeI$ taken at the normal emission angle at room temperature (a) and at low temperature (b). Red circles represent raw photoemission data, while the black line shows the fit result. The spectra are fitted with peaks of Voigt line shape (blue and green lines), asymmetric Gauss-Lorentz product for the Fermi level (grey line) and linear background (red line). FWHM was determined by the fitting of the spectrum far from the $\bar{\Gamma}$-point for each ARPES image.

Determination of the gap value at the $\bar{\Gamma}$-point was carried out by the fitting of the EDC and MDC curves. Fig. 2Sa and b present the EDC profiles and fit results at the $\bar{\Gamma}$-point of the ARPES images respectively shown in Fig. 1c and f. At room temperature (Fig. 2Sa and Fig. 1c of paper), the EDC profile contains only one peak indicative of the gap absence. After the cooling (Fig. 2Sb and Fig. 1f of the manuscript), the spectrum is found to consist of two pronounced peaks (shown in blue and green in Fig. 2Sb) with a gap of 90±10 meV separating them. Besides, the increased intensity at the Fermi level is seen, which can be due to the angular broadening during the photoemission measurements, tails of the bulk states or adsorption of the residual gas molecules.

Notably, there is a non-zero intensity within the gap which, as we show here, originates from the tails of the broadened bands. For the clear visualization, in Fig. 3S the ARPES data at 20 K (the same as in Fig.1f of the paper) are presented in comparison with the model dispersion relations broadened with Gaussian line shape. Images with the model dispersion are obtained using Eq. (2) of the paper with 2Δ=90 meV. Fig3Sb shows the image with FWHM of 0.1 eV, which is close to the experimental value. The intensity inside the gap is non-zero and has the distribution, similar to the experimental data (Fig.3Sa). In contrast, if the FWHM is set to 0.04 eV (Fig.3Sc) one can see a drop of the intensity within the gap, corresponding to a zero density of states.

In the V-doped BiTeI the reason of the bands broadening is twofold. The first factor is simply a randomness, while the second one is related to inhomogeneity of the 2DEG Rashba-split state coupling to the doped-in magnetic moments. Indeed, as it is shown in a recent scanning tunneling spectroscopy study [SR2], performed on the $Cr_x(Bi_{1-y}Sb_y)_{2-x}Te_3(0001)$, the inhomogeneous distribution of the *magnetic dopants* gives rise to a fluctuation of the exchange gap size throughout the surface. Therefore, one can expect the exchange gap of the V-doped BiTeI to show a size distribution across the surface as well, which also contributes to the line-width of the surface bands measured by ARPES.

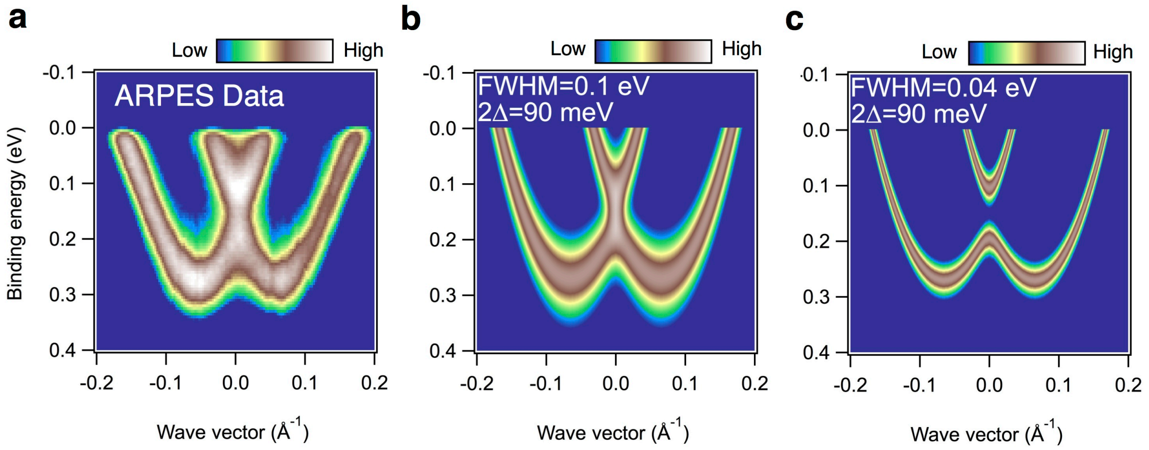

Fig. 3S (a) – ARPES spectrum of $Bi_{0.985}V_{0.015}TeI$ at 20 K, the same as in Fig.1f of the paper. Model dispersion relations obtained using Eq.(2) of the main text and the Gaussian broadening with FWHM of 0.1 eV (b) and 0.04 eV (c)

Fig.4S presents the photoemission spectrum taken at the normal emission at 20 K fitted with various parameters. One can see that the perfect fit can be obtained with using of Shirley background and addition of the weak peak at the BE of ~0.27 eV (thin red line). This peak can be related to the tails of the states from the I-terminated terraces. Nevertheless, such a variation of the fit parameters does not affect the value of the gap of 90±10 meV.

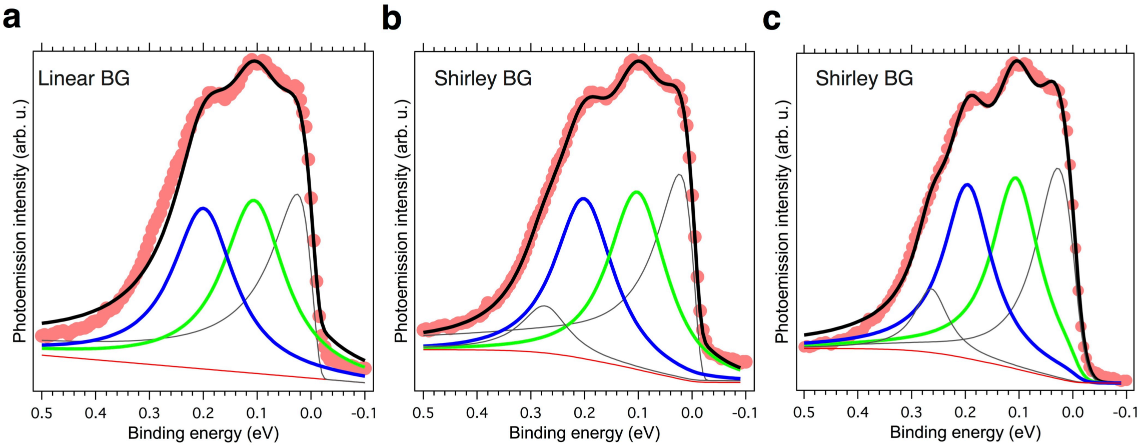

Fig. 4S Decomposition of the photoemission spectrum of $Bi_{0.985}V_{0.015}TeI$ taken at the normal emission angle at low temperature. Red circles represent raw photoemission data, while the black line shows the fit result. The spectra are fitted with peaks of Voigt line shape (blue and green lines) and (a) a linear background (red line) or (b,c) Shirley background. The peak near Fermi level (grey) is treated by the asymmetric Gauss-Lorentz product (a,b) and Lorentz-Fermi function product (c). FWHM was determined by the fitting of the spectrum far from the $\bar{\Gamma}$-point for each ARPES image.

Supplementary Note 3: Magnetic ordering of the V-doped BiTeI in the bulk and the Te-terminated surface

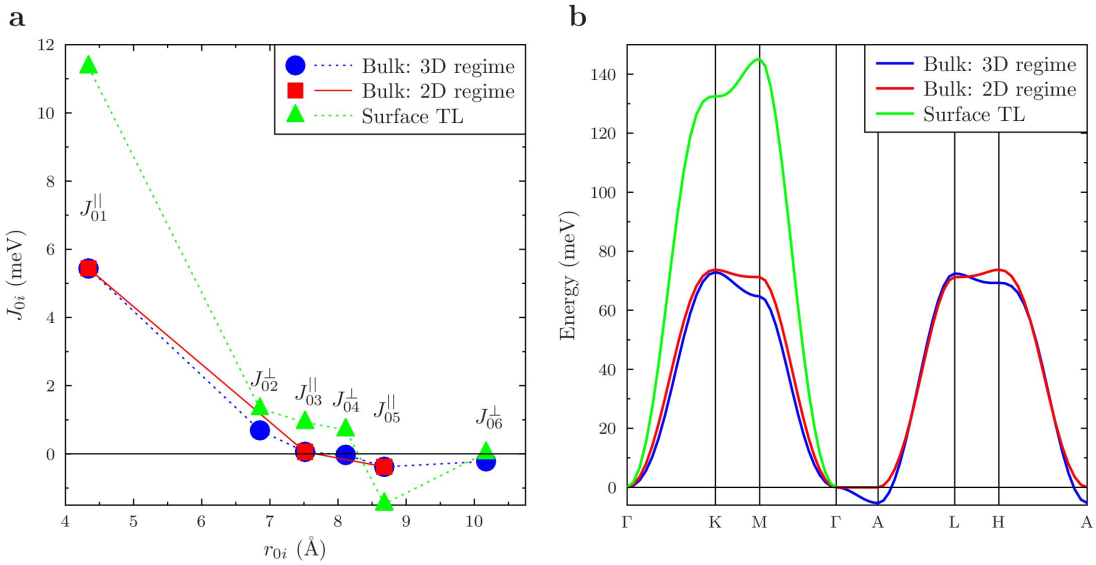

Fig. 5S**.** Exchange coupling constants J_{0i} (in meV) as a function of the V-V distance r_{0i} (a) and magnon dispersions (b) calculated for the bulk $Bi_{0.975}V_{0.025}TeI$ in the 3D (blue) and 2D (red) coupling regimes as well as for the topmost TL of the $Bi_{0.975}V_{0.025}TeI$ Te-terminated surface (green). In (a), the lines are guides to eyes. The ∥ and ⊥ superscripts distinguish exchange interactions between V atoms belonging to the same TL or the adjacent ones, respectively.

To characterize magnetic properties of the V-doped BiTeI we have performed additional theoretical and experimental studies. With a first-principles Green function method, we have computed parameters of the exchange coupling between the V local moments using the magnetic force theorem [SR3]. Once calculated, they have further been used for obtaining spin-wave spectra by diagonalization of the Heisenberg Hamiltonian and estimation of the magnetic transition temperature within the random-phase approximation. Such an approach has previously proven to be reliable for systems similar to the V-doped BiTeI – diluted magnetic semiconductors, magnetic oxides and magnetically-doped topological insulators [SR4-SR8].

First, we have studied a bulk V-doped BiTeI. To illustrate the issue of the $Bi_{1-x}V_xTeI$ bulk magnetism we have chosen a moderate concentration level, $x = 0.025$, which can also be representative of magnetic properties of the material with slightly smaller and higher x values. The exchange coupling parameters and magnon spectra, calculated for the ferromagnetic (FM) state in the bulk $Bi_{0.975}V_{0.025}TeI$ for the three-dimensional (3D) and two-dimensional (2D) coupling regimes are shown in Fig. 5S. For the 3D coupling case, when both intra- and inter-layer exchange interactions are taken into account, the results are shown by blue circles and blue line in the panels (a) and (b), respectively. In this case, a 3D ferromagnetic order turns out to be unstable as is evidenced by the negative energies that the spin-wave spectrum features along Γ-A, A-L and H-A directions (see Fig. 5Sb). In principle, this instability can be caused by both intra- ($J_{05}^{\parallel}$) and interlayer ($J_{04}^{\perp}$ and $J_{06}^{\perp}$) interactions since the exchange parameters describing them are negative (Fig. 5Sa). To determine whether the intralayer or the interlayer exchange is responsible for this instability we have artificially switched off the interlayer interactions, i.e. those described by the $J_{0i}^{\perp}$ parameters, $i = 2$, 4 and 6. We find that in this 2D coupling regime the magnons become completely positive along the in-plane directions A-L and H-A (see the red curve in Fig. 5Sb), what points to the interlayer interactions as a source of the instability. It should be noted, that the spin-wave spectrum is dispersionless along the vertical direction Γ-A which is a consequence of the absence of the interaction along z dimension. These results show that although a three-dimensional ferromagnetic order in $Bi_{0.975}V_{0.025}TeI$ is unstable, each triple layer (TL) on its own is ferromagnetic up to a temperature of about 9 K. Below 4 K, there is an interlayer antiferromagnetic ordering between the FM-ordered TLs in the bulk of the V-doped BiTeI. It appears due to the interlayer interactions described by the negative parameters $J_{04}^{\perp}$

and $J_{06}^{\perp}$ (Fig. 5Sa), that are responsible for the A-point minimum of the magnon mode at negative energy (Fig. 5Sb). However, at the Te-terminated surface, where the Rashba-split 2DEG state resides, the exchange interactions are significantly stronger than inside the bulk. As one can see in Fig. 5Sa, the magnitudes of the $J_{01}^{\parallel}$, $J_{03}^{\parallel}$ and $J_{05}^{\parallel}$ exchange parameters increase by the factors of two, nine and four, respectively. As a consequence, the magnon mode (green line) becomes harder along Γ-K and Γ-M directions, with a maximum at the M-point that is roughly twice as large as in the bulk case. This enhances the ordering temperature in the surface triple layer up to 28 K for $Bi_{0.975}V_{0.025}TeI$. Thus, it is the topmost FM triple layer that is responsible for the giant exchange gap opening at the V-doped BiTeI surface.

Supplementary Note 4: Slab thickness dependence of the Rashba parameter

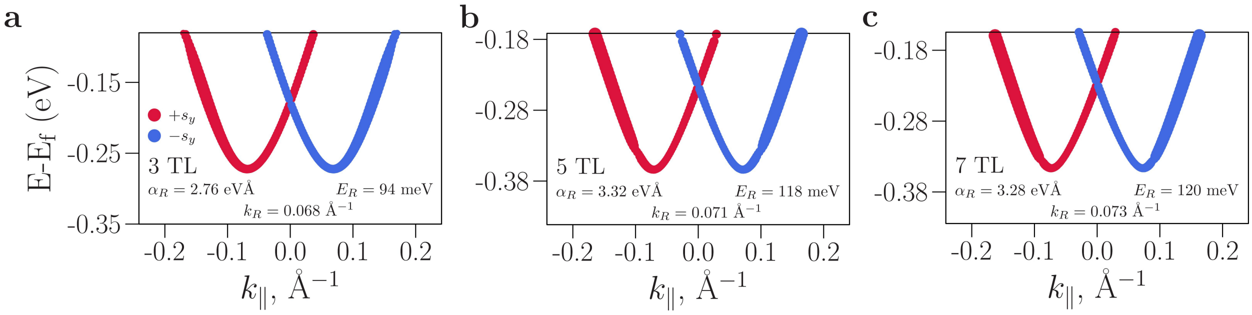

Fig. 6S Evolution of the DFT-calculated Rashba-split surface state of undoped BiTeI with Te termination for slab thickness 3 TLs (a), 5 TLs (b), and 7 TLs (c). Only $\pm s_y$ components of the spin are shown. The E_R, k_R, and α_R are indicated in the fields of the figures.

Fig. 6S compares the calculated dispersion of the Rashba-split Te-terminated (0001) surface state of BiTeI for the 3, 5 and 7 TL-block slabs in the (1x1) in-plane supercell. It can be seen that at thickness of 3 TL blocks the surface state is not completely developed since its α_R is only equal to 2.76 eVÅ (c.f. α_R=3.5 eVÅ in [SR9], obtained for 8 TLs), which is due to the small E_R=94 meV (the k_R, being the minimum parabola position, almost doesn't change with thickness). For the 7 TL-thick film we obtain α_R=3.28 eVÅ in agreement with [SR9].

In our study of V-doped BiTeI we use a 3 TL-block slab and a (3x3) in-plane supercell. A not completely developed surface state at such a thickness is not expected to affect the results and conclusions of the present work. Indeed, the qualitative discrepancy between the theoretically calculated and experimentally measured dispersions of the *gapped* surface state of V-doped BiTeI could appear when the calculated E_R for the pristine BiTeI(0001) is much smaller than the exchange gap, which forms upon magnetic doping. In the extreme case, the lower edge of the exchange gap at the $\bar{\Gamma}$-point could have gone below the parabolic minimums leading to the loss of the characteristic Rashba-like shape. As a result, the originally Rashba-split surface state would simply appear like two exchange-split parabolas, although with a Rashba-like spin texture. However, in the case of the 3 TL-block-thick slab E_R=94 meV which roughly equals to the experimentally measured magnitude of the exchange gap, which excludes appearance of the described scenario.

Supplementary Note 5: Details of the V-doped BiTeI(0001) surface bandstructure

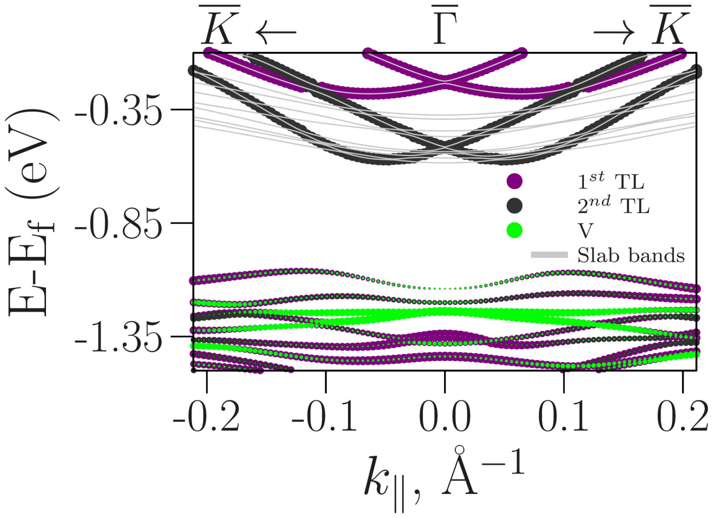

Fig. 7S The Te-terminated surface bandstructure of the V-doped BiTeI(0001). The size of purple, gray and green circles reflects the weight of the wave function on the topmost (1st) TL, subsurface (2nd) TL, and a vanadium site, respectively. Light gray lines show the bands as calculated for the 3 TL-thick slab.

Fig. 7S sheds light on the appearance of the artificial discontinuity of the outer branches of the Rashba-exchange-split state of the V-doped BiTeI(0001) (cf. Fig. 3b of the paper). Vanadium substitution into the subsurface Bi layer results in the *p*-doping whereby the states localized in the topmost TL experience an upward shift. In particular, this happens to the Rashba-exchange-split surface state, highlighted by purple in Fig. 7S. This leads to the situation when the quantum well state (gray color; the state is Rashba-split as well), localized in the subsurface TL, gets located below the Rashba-exchange-split surface state. The avoided crossing of the two states results in the discontinuity which is absent in the experiment since, contrary to the model used in the calculation (See Methods Section of the paper), each TL of our sample contains V dopants.

Supplementary Note 6: DFT-calculated surface band structure of V-doped BiTeI(0001) in the presence of Bi vacancy

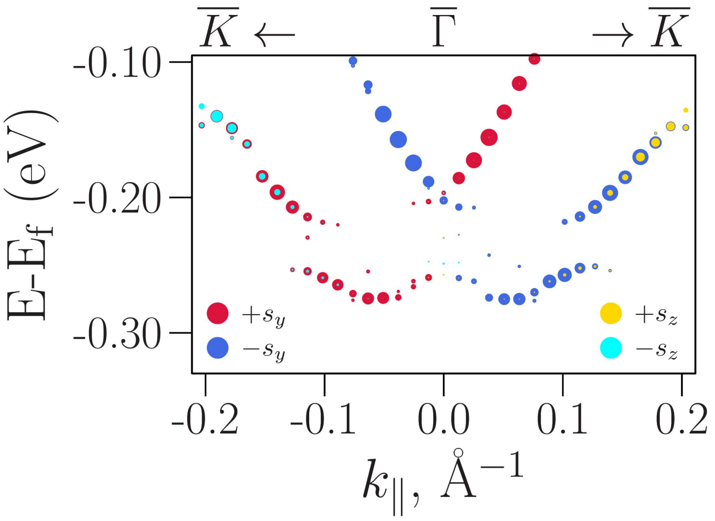

Fig. 8S Spin-resolved DFT-calculated surface bandstructure of the V-doped BiTeI(0001) in the presence of the Bi_{vac} (the projection of the wave function onto the Te-terminated surface TL block is shown). For the clear comparison, we shift E_F to the position corresponding to the undoped case without defects (Fig. 3a of the paper).

Figure 8S shows the Rashba-exchange-split surface state of V-doped BiTeI(0001) in the presence of the Bi_{vac}. Note that the calculation was performed for the slab comprised of 6TL blocks since the picture obtained for the thickness of 3 TLs was inconclusive. The exchange gap at the $\bar{\Gamma}$-point seems to be larger than that in the absence of the Bi vacancy (c.f. Fig. 3b of the paper). Nevertheless, from the data displayed in the figure, it appears to be difficult to unambiguously determine the magnitude of the exchange $\bar{\Gamma}$-point gap because its edges aren't well defined, as it was in the case of the V-doping solely (see Fig. 3b of the paper). The origin of the gap edges broadening is a strong hybridization of the 2DEG Rashba-split surface state with other slab-derived bands. It appears due to the state shift upon Bi vacancy introduction and is enhanced because of the final thickness of the slab (6 triple layers). Therefore, we resort to the *ab-initio*-based tight-binding calculations to estimate the exchange gap size for this case (see the paper text and Methods Section for details). To reveal the effect of the additional magnetization supplied by Bi vacancies in a clear and tractable way, what turned out to be impossible within the supercell calculation, one needs to use an alternative model that would not suffer from the drawbacks of the supercell model. Indeed, the inclusion of the defects explicitly in the *ab initio* calculation and extraction of the parameters for the tight-binding calculations would lead to a result similar to that shown in Fig. 8S, i.e. hardly a conclusive one. Therefore, instead of introducing the defects directly, we rather took into account their effect, i.e. an additional magnetization. This has been done by introducing an additional Zeeman-like contribution to the Hamiltonian, whose values have been set to the exchange splittings of the Bi, Te and I *p*-states calculated *ab initio* in the presence of both V dopant and Bi vacancy.

Supplementary references

[SR1] A. Crepaldi, L. Moreschini, G. Autès, C. Tournier-Colletta, S. Moser, N. Virk, H. Berger, Ph. Bugnon, Y. J. Chang, K. Kern, A. Bostwick, E. Rotenberg, O. V. Yazyev, and M. Grioni, Giant Ambipolar Rashba Effect in the Semiconductor BiTeI. Phys. Rev. Lett. **109**, 096803 (2012)

[SR2] I. Lee, C. K. Kim, J. Lee, S. J. L. Billinge, R. Zhong, J. A. Schneeloch, T. Liu, T. Valla, J. M. Tranquada, G. Gu, and J. C. Séamus Davis. Imaging Dirac-mass disorder from magnetic dopant atoms in the ferromagnetic topological insulator $Cr_x(Bi_{0.1}Sb_{0.9})_{2-x}Te_3$. Proceedings of the National Academy of Sciences **112**, 1316 (2015).

[SR3] A. I. Liechtenstein, M. I. Katsnelson, V. P. Antropov, and V. A. Gubanov, J. Magn. Magn. Mater. **67**, 65 (1987)

[SR4] L. V. Bekenov, V. N. Antonov, S. Ostanin, A. N. Yaresko, I. V. Maznichenko, W. Hergert, I. Mertig, and A. Ernst. Electronic and magnetic properties of $(Zn_{1-x}V_x)O$ diluted magnetic semiconductors elucidated from x-ray magnetic circular dichroism at V $L_{2,3}$ edges and first-principles calculations. Phys. Rev. B **84**, 134421 (2011).

[SR5] S. Ostanin, A. Ernst, L. M. Sandratskii, P. Bruno, M. Däne, I. D. Hughes, J. B. Staunton, W. Hergert, I. Mertig, and J. Kudrnovský. Mn-Stabilized Zirconia: From Imitation Diamonds to a New Potential High-TC Ferromagnetic Spintronics Material. Phys. Rev. Lett. **98**, 016101 (2007).

[SR6] G. Fischer, M. Däne, A. Ernst, P. Bruno, M. Lüders, Z. Szotek, W. Temmerman, and W. Hergert. Exchange coupling in transition metal monoxides: Electronic structure calculations. Phys. Rev. B **80**, 014408 (2009).

[SR7] J. Henk, A. Ernst, S. V. Eremeev, E. V. Chulkov, I. V. Maznichenko, and I. Mertig. Complex Spin Texture in the Pure and Mn-Doped Topological Insulator Bi_2Te_3. Phys. Rev. Lett. **108**, 206801 (2012).

[SR8] J. Henk, M. Flieger, I. V. Maznichenko, I. Mertig, A. Ernst, S. V. Eremeev, and E. V. Chulkov. Topological Character and Magnetism of the Dirac State in Mn-Doped Bi_2Te_3. Phys. Rev. Lett. **109**, 076801 (2012).

[SR9] S.V. Eremeev, I.A. Nechaev, E.V. Chulkov, Giant Rashba-type spin splitting at polar surfaces of BiTeI, Pis'ma v ZhETF **96**, 484 (2012)
